# Isolation and Synthesis of Laxaphycin B-Type Peptides: A Case Study and Clues to Their Biosynthesis

**DOI:** 10.3390/md13127065

**Published:** 2015-12-05

**Authors:** Louis Bornancin, France Boyaud, Zahia Mahiout, Isabelle Bonnard, Suzanne C. Mills, Bernard Banaigs, Nicolas Inguimbert

**Affiliations:** 1 Centre de Recherche Insulaire et Observatoire de l’Environnement (CRIOBE), USR 3278 EPHE-CNRS-UPVD, Université de Perpignan Via Domitia, 58 avenue P. Alduy, 66860 Perpignan, France; louis.bornancin@wanadoo.fr (L.B.); france.boyaud@gmail.com (F.B.); zahia.mahiout@hotmail.fr (Z.M.); isabelle.bonnard@univ-perp.fr (I.B.); 2 Centre de Recherche Insulaire et Observatoire de l’Environnement (CRIOBE), USR 3278 EPHE-CNRS-UPVD, BP 1013 Papetoai, Moorea, French Polynesia, France; suzanne.mills@univ-perp.fr; 3 Laboratoire d’Excellence “Corail”, 58, avenue Paul Alduy, 66860 Perpignan, France

**Keywords:** non ribosomal peptides, cyclic lipopeptide, solid phase peptide synthesis, *Anabaena*, *Lyngbya*

## Abstract

The laxaphyci’s B family constitutes a group of five related cyclic lipopeptides isolated from diverse cyanobacteria from all around the world. This group shares a typical structure of 12 amino acids from the l and d series, some of them hydroxylated at the beta position, and all containing a rare beta-amino decanoic acid. Nevertheless, they can be differentiated due to slight variations in the composition of their amino acids, but the configuration of their alpha carbon remains conserved. Here, we provide the synthesis and characterization of new laxaphycin B-type peptides. In doing so we discuss how the synthesis of laxaphycin B and analogues was developed. We also isolate minor acyclic laxaphycins B, which are considered clues to their biosynthesis.

## 1. Introduction

Among marine organisms, filamentous cyanobacteria occupy a special place and/or are of great interest for chemists because they produce a wide range of bioactive molecules, mainly cyclic lipopeptides [1,2,3]. Interestingly, they produce this class of secondary peptide metabolites via a non-ribosomal pathway that is responsible, for example, for the modification of natural amino acids into d-, *N*-methyl, β-hydroxylated, or dehydrated amino acids. These non-ribosomal peptide synthases (NRPS) are often associated with polyketide synthases (PKS) that allow fatty amino acids to be inserted within the peptide sequence [4,5]. The concomitant effects of these two multi-domain enzymes contribute to the vast diversity of structure observed in these secondary cyclopeptide metabolites [6].

Laxaphycins are cyclic lipopeptides synthesized through a hybrid PKS/NRPS biosynthetic pathway by different marine or freshwater cyanobacteria. They contain amino acids of alternate stereochemistry (l or d) and feature a rare fatty β-amino acid with a linear chain of up to 12 carbons [7]. Several studies have reported structural variants and likely biosynthetic derivatives of laxaphycins that can be separated into two groups, the laxaphycin A-type peptides, whichare cyclic undecapeptides, and the laxaphycin B-type peptides, which are cyclic dodecapeptides. Laxaphycin A-type and laxaphycin B-type peptides are generally found in the same cyanobacteria.

**Figure 1:**
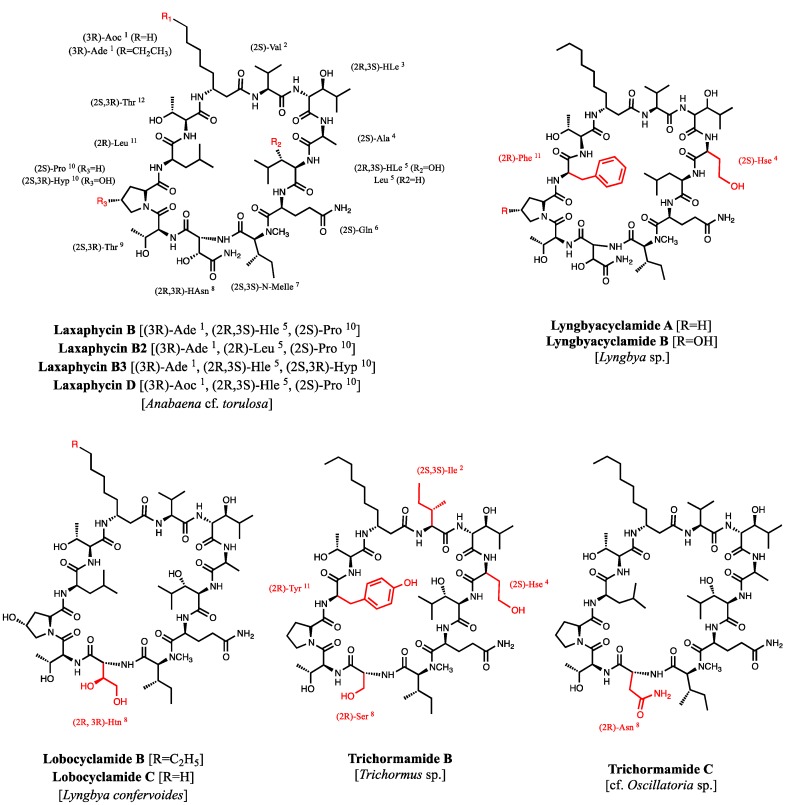
Laxaphycins B, B2, B3, and D and their analogs lyngbyacyclamides A–B, lobocyclamides B–C and trichormamides B–C. Differences between laxaphycins and their homologs are highlighted in red.

*Anabaena* *laxa* [8], *A. torulosa* [9], *Lyngbya confervoides* [10], *Trichormus* sp. [11], and *cf.* *Oscillatoria* sp. [12] express laxaphycins B, B2, B3, and D, lobocyclamides B and C, and trichormamides B and C. Furthermore a *Lyngbya* sp. strain produces lyngbyacyclamides A and B [13] (Figure 1). Horizontal gene transfer between cyanobacteria has been suggested as an explanation for the presence of all these closely related compounds in diverse species [14].

In the present study we were interested in the study of laxaphycin peptides from *A.* *cf.* *torulosa*, compounds that have already been found to reduce damage by consumers [15]. More specifically, we focus on both total synthesis and structural characterization of laxaphycin B-type peptides; total synthesis being the ultimate way to confirm or revise the proposed structure of such dodecapeptides possessing non-commercial amino acids.

We recently published the first total synthesis of laxaphycin B (short form: laxaB) and lyngbyacyclamide A [16]. Here we describe the synthesis of two new laxaB analogs, including the different steps and strategies that were taken to revise the initially reported structure. We also describe the structure of two new acyclic laxaB-type peptides. The presence of these acyclic dodecapeptides, named acyclolaxaphycin B (**11**) and acyclolaxaphycin B3 (**12**), together with the other laxaphycins in the extract of *A.* *cf.* *torulosa*, provide valuable information for the biosynthesis of laxaphycins. Understanding how these non-ribosomal peptides can be chemically synthesized and how such intriguing structures can be synthesized in nature is challenging.

## 2. Results and Discussion

### 2.1. Synthesis of Laxaphycin B Analogs

**Figure 2:**
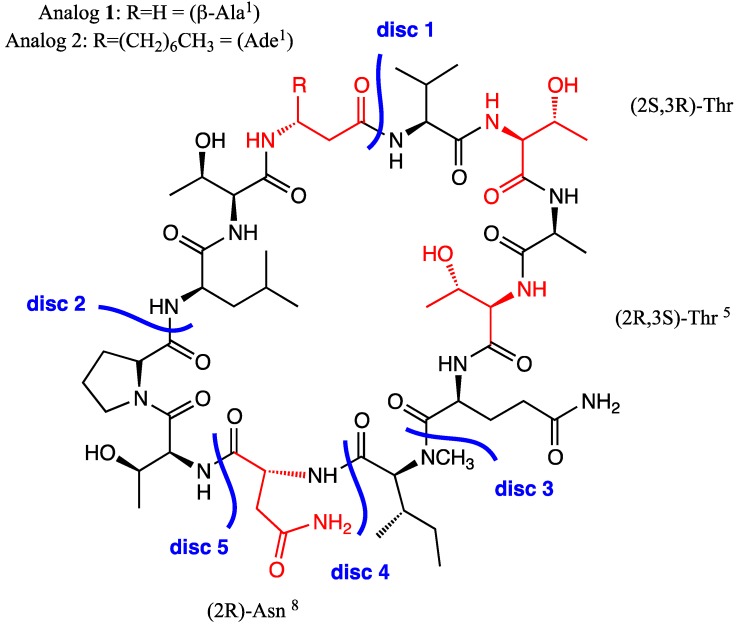
Proposed simplified analogs **1** and **2** of [(2*S*,3*S*)-Hle^3^]laxaphycin B and feasible disconnections.

The synthesis of lipocyclopeptides of marine origin is not straightforward, firstly because synthetic chemists have to make a choice among the different potential stereochemistry options for the isolated compounds. Secondly, they need to prepare non-natural amino acids and use them sparingly when developing the synthesis or even have recourse to use related analogs, before extending the synthesis to the natural compound. Taking these limitations into account, especially the uncertainty concerning the stereochemistry of the residue in position 3, we proposed an analog of the ((2*S*,3*S*)-Hle^3^)laxaphycin B, the compound initially described for laxaB, in which the 2-aminodecanoic acid (Ade), the 3-hydroxyleucines (Hle), and the 3-hydroxyasparagine (HAsn) were replaced by the simplest and commercially available β-alanine, threonine, and asparagine, respectively, leading to analog **1** (Figure 2). The replacement amino acids were selected with respect to the stereochemistry of the alpha carbon of the published compound. These peptides can be obtained either by adopting an in-solution synthetic strategy or by solid phase peptide synthesis (SPPS). Due to its numerous advantages including the avoidance of repetitive purification steps and flexibility, SPPS was used preferentially to the in-solution strategy. Furthermore, we make use of an instrumentation combining the advantages of automation and microwave heating that speeds up synthesis and increases overall yield and purity for difficult peptide sequences [17,18,19,20].

#### 2.1.1. Retrosynthetic Analysis of the Laxaphycin B Analog **1**

Five disconnections were envisaged for the cyclization of a linear precursor obtained by a Fmoc/*t*Bu SPPS strategy. Depending on the position of the disconnected amide bond, the cyclization can be performed in solution or on the resin [21]. Thus disconnections 1 and 2 (Figure 2) would imply the use of chlorotrityl resin and a subsequent cleavage in dilute acidic conditions of a completely protected linear peptide that has to be cyclized in solution in the last step. Firstly, this option was discarded because a long reaction time is prone to generate dimerization or oligomerization even in dilute conditions and this option requires sophisticated setups involving syringe pumps to deliver the reagents [22,23]. Secondly, the use of microwave irradiation during peptide elongation was recently shown to be deleterious because of the sensitivity of 2-Cl trityl-resin to extended heating periods [24]. Subsequently, we considered the side chains of an aspartic or a glutamic acid as possible anchoring points onto a low loading Rink amide resin since the final cleavage from the resin would produce the corresponding asparagine or glutamine. Indeed, this would allow the entire synthesis to be performed on the resin due to the temporary allyl protection of the C-terminal α-carboxyl-group [25]. Direct use of glutamic acid was inconvenient since the ultimate step of the synthesis, the cyclization, would occur at an impractical coupling site between its carboxylic function and the *N*-methylated group of the isoleucine (disconnection 3, Figure 2).

**Figure 3:**
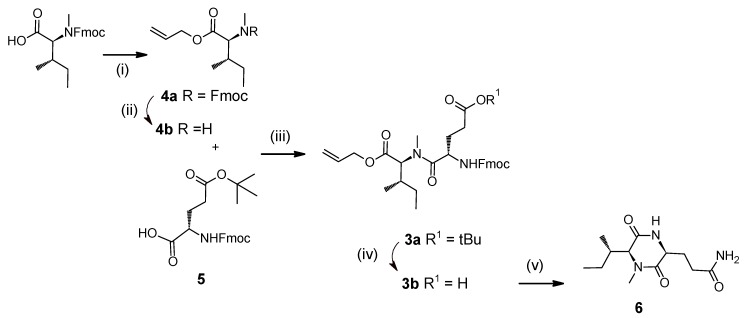
Proposed dipeptide **3b** as a starting material for the synthesis of ((2*S*,3*S*)-Hle^3^)laxaphycin B analogs and formation of undesired diketopiperazine **6**. Reagents and conditions: (i) Cs(CO_3_)_2_, DMF, Allylbromide, 76%; (ii) piperidine, 70%; (iii) DIEA, COMU, DMF, 70%; (iv) TFA, CH_2_Cl_2_; (v) Rink amide resin, HATU, DIEA, piperidine.

Alternatively, we proposed grafting the FmocGlu-*N*-MeIle(Oallyl) dipeptide (**3b**) on the resin (Figure 2, disconnection 4) that was conveniently prepared by successively coupling compounds **4** and **5** using COMU [26] with final deprotection under acidic conditions of the tertiobutylester (Figure 3). Unfortunately, after the addition of the dipeptide onto the resin support and deprotection of the Fmoc protective group, diketopiperazine **6** forms immediately and precludes further extension of the peptide [27]. This was confirmed after acidic release of compound **6** from the resin, and analysis of the crude by LC-MS, which showed a major peak with the expected mass [M + H]^+^ *m*/*z* 256.

Finally, among the remaining possibilities our choice was dictated by the presence of the non-commercially available (2*R*,3*R*) β-OH-Asn^8^, a constituent of natural laxaphycin B. In order to test its potential for future use, we had to develop a protocol for its synthesis starting from a surrogate, thus (2*R*)-aspartic acid was chosen as an anchoring point to the resin (disconnection 5). It is noteworthy that one advantage of this strategy resides in the possibility of accessing trichormamide C analogues.

#### 2.1.2. Solid Phase Synthesis Preliminary Assay of ((2*S*,3*S*)-Hle^3^)laxaphycin B Analogs

**Figure 4:**
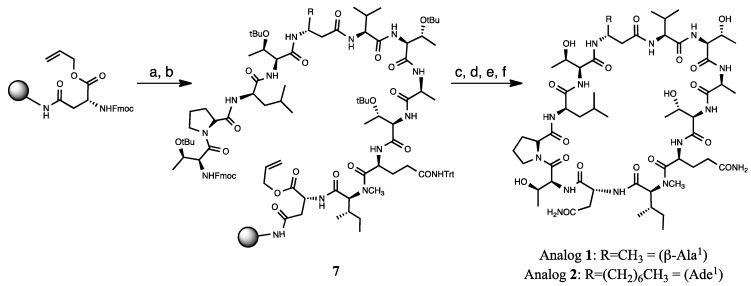
Syntheses of the laxaphycin B analogs **1** and **2**. Reagents and conditions: (**a**): (i) 20% *v*/*v* piperidine, DMF (ii) Fmoc-*N*-MeIle, HATU, DMF, MW 25 W, 70 °C, 5 min; (**b**) repetition of conditions (i) and (ii) for Fmoc-Gln-(Trt) 2 × 10 min, Fmoc-d-Thr(tBu)-OH, Fmoc-Ala-OH, Fmoc-Thr(tBu)-OH, Fmoc-Val-OH, Fmoc-β-Ala-OH or Fmoc Ade 2 × 10 min, Fmoc-Thr(tBu)-OH, Fmoc-d-Leu-OH, Fmoc-Pro-OH, Fmoc-Thr(tBu)-OH; (**c**) Pd(PPh_3_)_4_, CHCl_3_/AcOH/NMM, 3.7/0.2/0.1, *v*/*v*/*v* (**d**) 20% *v*/*v* pipéridine, DMF, rt, 2 × 2 min (e) DIC, oxyma, DMF, MW 25 W, 70 °C 3 × 15 min; (**f**) TFA/TIS/H2O 9.5/0.25/0.25, *v*/*v*/*v*, TA, 3 h.

The ((2*S*,3*S*)-Hle^3^)laxaphycin B analog **1** was assembled by stepwise SPPS starting with the introduction of the *N*-Fmoc-aspartic acid α-allyl ester onto a low loading rink amide MBHA resin (0.36 mmol/g) using the effective 2-(7-aza-1H-benzotriazole-1-yl)-1,1,3,3-tetramethyl uronium hexaflurorophosphate HATU as a coupling reagent and a fivefold excess of standard amino acids with regard to resin capacity (Figure 4). The choice of this relatively expensive reagent was guided by preliminary results, which revealed the presence of a difficult coupling site located at the Gln and *N*-Me-Ile junction, requiring a triple coupling and a capping step. After synthesis of the linear precursor **7**, the α-carbonyl allyl protecting group of Asn was removed using Pd(PPh_3_)_4_. Head to tail cyclization of the resin-bound peptide was accomplished after Fmoc removal using DIC/oxyma (3 × 15 min), a base-free condition known to reduce epimerization [28]. Final acidic cleavage followed by HPLC-purification produced the desired peptide **1** (Table 1 and Table S1).

**Table 1:** Retention time (Rt) in minutes and observed mass peaks of the analogs **1**, **2**, **8**, and laxaB.

Compound	1	2	((2 *S*,3*S*)-Hleu^3^)laxa B (8)	Laxaphycin B (9)
**Mass Peaks**	[M + H]^+^ *m*/*z* 1225	[M + H]^+^ *m*/*z* 1323	[M + H]^+^ *m*/*z* 1395	[M + H]^+^ *m*/*z* 1395
**Rt (min)**	8.64	16.01	16.3	Natural: 14.8
				Synthetic: 14.8

The next step on the way to laxaphycin B synthesis was the introduction of the (2*R*)-2-aminodecanoic acid (Ade) obtained using an already described procedure and subsequent protection with a Fmoc group [29]. Indeed, due to the high hydrophobic character of both Ade and the peptide itself, aggregation of the peptide growing chain was expected even if counterbalanced by microwave irradiation [17]. As expected, a drop in the coupling efficacy of Ade was observed by UV monitoring of the Fmoc group deprotection and was corrected for with a double coupling procedure. Thus analog **2** was obtained and characterized by ^1^H-NMR and LC-MS analysis (Table 1 and Table S1).

^1^H-NMR COSY experiments allowed the chemical shift of all amino acids to be assigned, but ambiguity remained for the four threonines that could not be distinguished. Nevertheless, a comparison of NMR spectra of the closely related analogs **1** and **2** revealed considerable consistency in the observed chemical shift (Figures S1 and S2).

#### 2.1.3. Laxaphycin B Revised Structure

Finally, as recently reported, the above synthesis method was applied to laxaB [16]. Briefly, after introduction of the rare (2*R*,3*R*)-Fmoc-β-hydroxyaspartic α-allyl ester, (2R,3S)-Fmoc-Hle-(OTBDMS)-OH and (2*S*,3*S*)-Fmoc-Hle-(OTBDMS)-OH, 3 mg of pure peptide (**8**) was obtained [30,31]. The recorded molecular mass of [M + H]^+^ *m*/*z* 1395 and [M + Na]^+^ *m*/*z* 1417 from LC-MS ESI+ analysis corresponds to the one expected for laxaB. Nevertheless, a comparison of the retention time of compound (**8**) with a natural sample of laxaB (**9**) revealed differences that were further confirmed by co-injection (Table 1) and comparison of NMR spectra. Thus, we could conclude from this experiment that the synthesized compound (**8**) is a diastereoisomer of laxaB (**9**). In a last effort to obtain the natural compound, we hypothesized that the hydroxyleucines must have the same configuration as observed for lobocyclamide B. Repetition of the developed synthesis method using (2*R*,3*S*)-Fmoc-Hle-(OTBDMS)-OH produced, after purification, 3 mg of peptide **9** co-eluting with laxaphycin B and presenting the same ^1^H-NMR and mass spectra as the natural compound.

### 2.2. New Natural Acyclolaxaphycin B Analogs

Collection and extraction of *A.* cf *torulosa* and initial separation of the organic extract were described in a previous paper [9]. Further examination of the more polar flash chromatography fractions obtained from the organic extract, by C18 RP HPLC yielded two HPLC pure peaks, acyclolaxaphycin B (short form: acyclolaxaB) and acyclolaxaphycin B3 (short form: acyclolaxaB3). AcyclolaxaB (2 mg) and acyclolaxaB3 (3 mg) were obtained as colorless amorphous solids and responded positively to a ninhydrin test suggesting a non-blocked *N*-terminus. LC-MS analysis of pure compounds with electrospray positive ionization revealed two different peptides whose *m*/*z* values are 18 units higher than both laxaphycins B (**9**) and B3 (**10)**.

#### 2.2.1. Acyclolaxaphycin B (**11**): Structure Elucidation

High-resolution electrospray ionization mass spectrometry (HRESIMS) analysis yielded an [M + H]^+^ pseudomolecular ion at *m*/*z* 1413.8595 for a molecular formula of C_65_H_116_N_14_O_20_ that was supported by NMR spectroscopic analysis. A comparison with laxaphycin B (C_65_H_114_N_14_O_19_) revealed that this corresponds to a gain of H_2_O.

In the ^1^H-NMR spectrum of acyclolaxaB (**11**), recorded at 500 MHz in DMSO-*d*_6_, the close structural relationship between the two peptides was clear; the spectrum exhibited, in the NH proton region, signals typical for CONH_2_ protons corresponding to Gln (2 bs, δ_H_6.79, and δ_H_7.14) and Asn (2 bs, δ_H_7.26, and δ_H_7.30) similar to those observed for laxaB (Figure S3). Only one significant difference was found in the NH proton region: nine NH doublets and one large singlet (2H) were visible in acyclolaxaB ^1^H-NMR spectrum, instead of the 10 NH doublets observed between 7.4 and 8.4 ppm for laxaB.

Almost all ^1^H and ^13^C resonances of acyclolaxaB (Table 2) could be assigned using extensive 2D NMR analysis including COSY, TOCSY, HSQC, HSQC-TOCSY, and ROESY (Figures S4–S7).

Initially, spin systems in TOCSY spectrum were identified starting from the signals of the backbone amide protons in the region 8.5 to 6.5 ppm. From the characteristic chemical shift and comparison with laxaB, eight amino acids could be identified as Hle (2×), Gln, Val, Leu, Thr (2×), and HAsn. A β-Ade residue system was identified starting from a doublet at 7.53 ppm and possessing an AA’BB’ spin system (2.27 and 2.38 ppm) with additional signals at 4.05, 1.34–1.40, then 1.20–1.23 ppm. One spin system lacking an amide proton was identified as *N*-MeIle due to the correlations of its Hα and Hβ at 4.71 and 1.91 ppm, respectively. One last amino acid, attributed to Ala residue, was identified starting from a broad singlet (two protons) at 8.04 ppm, to Hα (δ_H_4.03, 1H, overlapped bs) and Hβ ( δ_H_1.36, 3H, d).

**Table 2:** ^1^H and ^13^C NMR data for laxaphycins B and B3 and acyclolaxaphycins B and B3 in DMSO-*d_6_*.

	Laxaphycin B		Acyclolaxaphycin B		Laxaphycin B3		Acyclolaxaphycin B3	
	^13^C	^1^H	^13^C	^1^H	^13^C	^1^H	^13^C	^1^H
β Ade^1^								
NH	-	7.58	-	7.53	-	7.52	-	7.53
CαH_2_	40.28	2.33/2.40	40.52	2.27/2.38	-	2.30/2.44	40.42	2.28/2.40
CβH	45.93	4.11	46.27	4.05	45.92	4.08	46.13	4.05
CγH_2_	33.45	1.29/1.40	33.62	1.34/1.40	33.41	1.40	33.49	1.33/1.40
CδH_2_	28.67 *	1.24	28.77	1.23	28.69 *	1.24	28.69	1.21
CεH_2_	28.47 *	1.20	28.61	1.20	28.47 *	1.20	28.53	1.21
CζH_2_	25.18 *	1.20	25.28	1.21	25.22 *	1.20	25.20	1.22
CηH_2_	31.11 *	1.20	31.22	1.21	31.10 *	1.20	31.15	1.20
CθH_2_	21.92 *	1.20	22.04	1.20	21.92 *	1.20	21.97	1.24
CιH_3_	13.79	0.84	13.91	0.85	13.79	0.82	13.81	0.83
CO	171.14	-	170.30	-	171.30	-	170.15	-
Val^2^								
NH	-	8.18	-	7.89	-	8.10	-	7.89
CαH	59.03	4.09	57.64	4.30	58.89	4.12	57.50	4.31
CβH_2_	29.33	1.97	30.59	2.02	29.37	1.98	30.51	2.02
CγH_3_	18.80	0.91	18.85	0.93	18.56	0.88	18.89	0.93
Cγ'H_3_	18.87	0.85	18.95	0.81	18.85	0.84	18.78	0.81
CO	171.05	-	171.27	-	171.30	-	171.15	-
HLe^3^								
NH	-	7.94		7.69	-	7.90	-	7.70
CαH	55.23	4.34	54.30	4.44	55.15	4.37	54.21	4.44
CβH	76.37	3.49	76.06	3.53	76.48	3.50	76.13	3.53
OH	-	4.94	-	—	-	4.90	-	—
CγH	30.54	1.58	30.68	1.51	30.57	1.60	30.84	1.52
CδH_3_	19.22 *	0.89	19.19	0.91	18.76 *	0.89	19.23	0.91
Cδ'H_3_	18.56	0.76	18.74	0.76	18.43	0.76	18.67	0.76
CO	171.35	-	172.40	-	-	-	172.34	-
Ala^4^								
NH/NH_2_	-	7.86	-	8.04	-	7.87	-	8.05
CαH	49.28	4.22	48.30	4.03	49.30	4.22	48.20	4.04
CβH_3_	17.55	1.31	17.43	1.36	17.65	1.32	17.38	1.36
CO	172.33	-	170.02	-	172.47	-	169.87	-
HLe^5^								
NH	-	7.69	-	8.34	-	7.61	-	8.37
CαH	55.52	4.28	55.40	4.44	55.64	4.28	55.27	4.46
CβH	75.80	3.49	76.21	3.53	75.78	3.48	75.94	3.53
OH	-	5.03	-	—	-	5.05	-	—
CγH	29.90	1.56	30.65	1.51	29.84	1.58	30.73	1.51
CδH_3_	18.65 *	0.89	17.58	0.82	18.69 *	0.88	19.14	0.83
Cδ'H_3_	18.56	0.76	19.28	0.81	-	0.74	17.52	0.83
CO	170.50	-	169.74	-	170.60	-	169.64	-
Gln^6^								
NH	-	7.77	-	8.02	-	7.56	-	8.04
CαH	49.16	4.63	48.94	4.69	49.40	4.58	48.78	4.70
CβH_2_	26.39	1.75/1.97	26.90	1.76/1.93	-	1.64/2.00	26.86	1.77/1.94
CγH_2_	30.72	2.04/2.10	30.63	2.12	-	2.15/2.23	30.70	2.13
CON	174.60	-	174.38	-	174.74	-	174.31	-
NH_2_	-	6.85/7.22	-	6.79/7.14	-	6.79/7.17	-	6.80/7.16
CO	172.49	-	172.45	-	172.64	-	172.27	-
*N*-MeIle^7^								
NCH_3_	30.03	2.97	30.24	2.97	30.15	3.01	30.20	2.98
CαH	59.85	4.72	59.94	4.71	59.87	4.73	59.79	4.73
CβH	31.56	1.90	31.50	1.91	31.80	1.90	31.41	1.92
CγH_2_	23.88	0.89/1.29	23.98	0.87/1.28	-	0.74/1.27	23.92	0.87/1.27
Cγ'H_3_	15.08	0.76	15.25	0.78	14.99	0.74	15.21	0.79
CδH_3_	10.33	0.78	10.48	0.79	10.31	0.75	10.41	0.78
CO	170.02	-	169.66	-	170.10	-	169.47	-
HAsn^8^								
NH	-	7.64	-	7.41	-	7.66	-	7.41
CαH	55.52	4.63	55.22	4.67	55.53	4.63	55.13	4.71
CβH	70.44	4.31	71.04	4.36	70.33	4.35	70.99	4.37
OH	-	5.79	-	5.78	-	5.70	-	-
CON	173.37	-	173.20	-	173.37	-	173.20	-
NH_2_	-	7.27	-	7.26/7.30	-	7.17	-	7.27/7.32
CO	169.16	-	168.92	-	169.12	-	168.75	-
Thr^9^								
NH	-	7.33	-	7.63	-	7.12	-	7.63
CαH	55.61	4.49	55.25	4.57	55.83	4.46	55.56	4.56
CβH	66.23	3.93	66.54	3.98	66.43	3.90	66.50	3.97
OH	-	4.94	-	-	-	4.89	-	-
CγH_3_	18.87	1.05	18.64	1.05	18.85 *	1.03	18.59	1.05
CO	168.58	-	168.87 *	-	168.70 *	-	169.04 *	-
Pro^10^/Hyp^10^								
CαH	59.60	4.33	59.90	4.37	58.62	4.43	58.87	4.44
CβH_2_	29.08	1.82/2.04	28.77	1.83/2.03	37.73	1.84/2.01	37.45	1.89/2.05
CγH_2_	24.00	1.80/1.90	24.16	1.83/1.90	68.50	4.32	68.48	4.31
OH						5.08		-
CδH_2_	47.16	3.68	47.49	3.64/3.75	55.48	3.58/3.72	55.60	3.60/3.76
CO	171.21	-	171.42	-	171.47 **	-	171.33	-
Leu^11^								
NH	-	7.89	-	7.77	-	7.86	-	7.84
CαH	51.36	4.31	51.44	4.30	51.31	4.35	51.36	4.29
CβH_2_	40.82	1.47	40.44	1.47	41.24	1.47	40.51	1.46
CγH	24.06	1.53	24.09	1.58	24.12	1.52	24.06	1.58
CδH_3_	22.71	0.87	22.96	0.86	22.75	0.86	22.83	0.86
Cδ'H_3_	21.76	0.82	21.42	0.84	21.72	0.80	21.43	0.83
CO	171.67	-	171.83	-	171.41 **	-	171.33	-
Thr^12^								
NH	-	7.74	-	7.57	-	7.68	-	7.59
CαH	57.85	4.11	58.13	4.10	58.17	4.10	58.12	4.10
CβH	66.19	4.00	66.52	3.97	66.35	3.97	66.50	3.97
OH	-	4.78	-	-	-	4.80	-	-
CγH_3_	19.46	0.99	19.55	0.99	19.48	0.99	19.45	1.00
CO	168.67	-	168.87 *	-	168.67 *	-	168.99 *	-

*^,^** Thr^9^ and Thr^12^ Chemical shifts may be interchanged.

**Figure 5:**
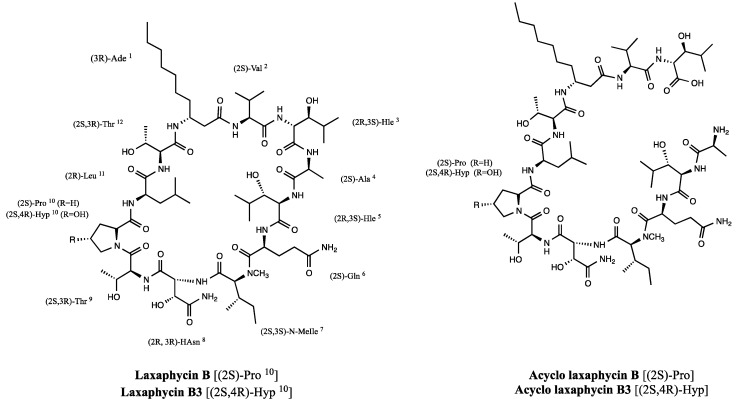
Structures of laxaphycins B (**9**) and B3 (**10**), and acyclolaxaphycins B (**11**) and B3 (**12**).

Sequence-specific assignments were determined from the HMBC correlations (Figure S8) between carbonyl carbons (residue i) and NH or NCH_3_ protons (residue i+1). These data suggested the presence of two fragments consisting of Ala-Hle-Gln-*N*-MeIle-HAsn-Thr (fragment 1) and Pro-Leu-Thr-β-Ade-Val-Hle (fragment 2). These two partial sequences were confirmed by ROESY correlations between Hα or Hβ (residue i) and NH or NCH_3_ (residue i+1). Fragments 1 and 2 were assembled by two inter-residue ROESY correlations between Hα (δ_H_4.57) and Hβ (δ_H_3.98) of Thr^9^ and Hδ (δ_H_3.64/3.75) of Pro^10^, establishing the complete sequence as Ala-Hle-Gln-*N*-MeIle-HAsn-Thr-Pro-Leu-Thr-β-Ade-Val-Hle (Figure 5). MS/MS data for **11** were consistent with the proposed amino acid sequence with the y ions at *m*/*z* 1213.50 (y10), 1085.58 (y9), 828.42 (y7), and 727.42 (y6) and the b ions at *m*/*z* 1266.75 (b11), 1167.58 (b10), and 456.25 (b4).

#### 2.2.2. Acyclolaxaphycin B3 (**12**): Structure Elucidation

Preliminary spectral data examination, including ^1^H and ^13^C-NMR spectroscopy, showed that the new compound was an analog of laxaB3 (**10**) and acyclolaxaB (**11**) (Figure S9). HRESIMS analysis yielded a [M + H]^+^ pseudomolecular ion at *m*/*z* 1429.8482 for a molecular formula of C_65_H_116_N_14_O_21_. In comparison to laxaB3 (C_65_H_114_N_14_O_20_), this corresponds to a gain of H_2_O and to acyclolaxaB gain of an oxygen atom.

A similar pattern of fragmentation for both compounds **11** and **12** was observed. Comparison of MS/MS spectra showed the same b4 fragment at *m*/*z* 456.25, the b11 (*m*/*z* 1282.76), y6 (*m*/*z* 743.49), y7 (*m*/*z* 844.54), y9 (*m*/*z* 1101.68), and y10 (*m*/*z* 1229.72) ions being shifted to a higher mass by 16 amu. In the HRESIMS/MS spectra of **12**, b9 (*m*/*z* 1014.55060, C_44_H_76_N_11_O_16_^+^, ∆obs/calc = 0.004) and b11 (*m*/*z* 1282.76672, C_59_H_104_N_13_O_18_^+^, ∆obs/calc = 0.005) fragments were observed. The y6 and b9 fragments in compound **12** shifted by 16 amu compared to **11**, suggesting that the variable residue could be in position 10, 11, or 12, corresponding to the Pro, Leu, or Thr residues, respectively.

The NMR spectral analysis (Figures S9–S14) of acyclolaxaB3 showed remarkable similarities with acyclolaxaB (**11**) and established the variable residue as Pro/Hyp (Figure 5). The significant difference was the presence of an additional hydroxyl group on proline (Hγ at 4.31 ppm *vs.* two Hγ at 1.83 and 1.90 ppm for compound **11**; Cγ at 68.48 ppm *vs.* 24.16 ppm for compound **11**; Cβ and Cδ were also deblinded by the presence of the hydroxyl function (∆δ 8.68 and 8.11 ppm, respectively)). HMBC and ROESY correlations established the complete sequence as Ala-Hle-Gln-*N*-MeIle-HAsn-Thr-Hyp-Leu-Thr-β-Ade-Val-Hle for compound **12**, and the gross structure of the new compound, acyclolaxaphycin B3, differed from acyclolaxaphycin B with a replacement of Pro by Hyp.

#### 2.2.3. Acyclolaxaphycins B (**11**) and B3 (**12**): Clues to Their Biosynthesis

Acyclolaxaphycins B (**11**) and B3 (**12**) are acyclic analogs of laxaphycins B (**9**) and B3 (**10**), respectively. They are two novel acyclic structural variants of a core structure (B-type laxaphycins) composed of about 10 cyanobacterial β-amino fatty acid cyclic dodecapeptides, laxaphycins B, B2, B3, and D, lyngbyacyclamides A–B, lobocyclamides B–C, and trichormamides B–C (Figure 1) with conserved amino acid residues. The chemical structure of all these compounds were similar, maintaining a 12-membered ring and sharing the (3*R*)-β-amino fatty acid (β-Aoc or β-Ade), (2*R*,3*S*)-Hle, (2*S*)-Gln, (2*S*)-*N*-MeIle, (2*S*,3*R*)-Thr, and (2*S*,3*R*)-Thr in the positions 1, 3, 6, 7, 9, and 12. In positions 2, 4, 5, 8, 10, and 11, the amino acid residues can vary, but their configuration at each position is strongly conserved.

An important subset of the β-hydroxylation of various amino acid residues observed for non-ribosomal synthesized peptides is catalyzed by cytochrome P450 monoxygenases [32,33,34]. The same biological machinery is certainly responsible for the β-hydroxylation of leucine and asparagine in laxaphycins and as this reaction is stereospecific, the stereochemistry of hydroxy-leucines, in position 3 and 5, and hydroxy-asparagine, in position 8, must be conserved.

Furthermore, both new peptides showed very similar NMR chemical shifts to laxaphycins B and B3 for the peptidic chain as well as for the side chains, indicating a conservation of the stereochemistry between cyclic and acyclic analogs. An indication of this homology could be seen in the comparison of ^1^H and ^13^C resonances of acyclolaxaphycins B and B3 with the parent compounds laxaphycins B and B3 (Hα, Hβ, Cα, and Cβ acyclolaxaphycins B and B3 resonances subtracted from the equivalent ones of laxaphycin B and B3, respectively). With the exception of the structurally modified parts of the molecule, residues 4 and 5 on the NH terminal side and residues 2 and 3 on the COOH terminal side, the maximum difference (∆δ) observed was less than 0.1 ppm in ^1^H and 0.8 ppm in ^13^C.

Therefore the complete structure of the two new compounds can be reasonably proposed as: (2*S*)-Ala—(2*R*,3*S*)-HLe—(2*S*)-Gln—(2*S*,3*S*)-*N*-MeIle—(2*R*,3*R*)-HAsn—(2*S*,3*R*)-Thr—(2*S*)-Pro—(2*R*)-Leu—(2*S*,3*R*)-Thr—(3*R*)-β-Ade—(2*S*)-Val—(2*R*,3*S*)-HLe for acyclolaxaphycin B (**11**) and (2*S*)-Ala—(2*R*,3*S*)-HLe—(2*S*)-Gln—(2*S*,3*S*)-*N*-MeIle—(2*R*,3*R*)-HAsn—(2*S*,3*R*)-Thr—(2S,4R)-4-Hyp—(2*R*)-Leu—(2*S*,3*R*)-Thr—(3*R*)-β-Ade—(2*S*)-Val—(2*R*,3*S*)-HLe for acyclolaxaphycin B3 (**12**).

A putative operon encoding the biosynthetic pathway for β-amino fatty acid lipopeptides, the puwainaphycins, was identified in the cyanobacterium *Cylindrospermum alatosporum*; the peptide biosynthesis process is initiated by the activation of a fatty acid residue via fatty acyl-AMP ligase (FAAL) and continued by a multidomain non-ribosomal peptide synthase/polyketide synthetase [35]. The last module incorporates a thioesterase domain in its terminal part that cleaves the finished puwainaphycin chain from the peptidyl carrier protein, thus promoting its cyclization between the NH_2_ of β-amino fatty acid and the COOH of a proline residue. The characterization of the two novel acyclic laxaphycin variants **11** and **12** with alanine as NH terminal and Hle as COOH terminal seemed to indicate that in the case of B-type laxaphycins, the biosynthesis process starts with NRPS modules instead of FAAL and acyl carrier protein (ACP) ligase, with the ring closure being performed through a cyclization reaction between the amino group of the alanine residue and the carbonyl of the hydroxyleucine residue. However, one cannot exclude that acyclolaxaphycins B and B3 are enzymatic degradation products formed during cyanobacteria blooms. Enzymatic degradation is often used in resistance mechanisms in the microbial world or in competitive interspecific interactions. Enzymes that degrade or modify natural products provide protection by decreasing toxicity or by regulating the signaling functions of metabolites. Recently, Hoefler *et al.* have observed hydrolysis of cyclic lipopeptides surfactins by bacterial competition using imaging mass spectrometry [36]. However, the ring opening of the cyclic surfactins occured at the ester functional group, which is not the case in laxaphycin peptides.

## 3. Experimental Section

### 3.1. Sampling Sites

The cyanobacterium *A.* cf *torulosa* was collected by SCUBA diving at a depth of 1–3 m in the Pacific Ocean, Moorea, French Polynesia. The cyanobacterium sample was sealed underwater in a bag with seawater and then freeze-dried.

### 3.2. Isolation Procedure

The freeze-dried sample of cynaobacterium A. cf torulosa (600 g) was extracted at room temperature three times with a mixture of MeOH-CH_2_Cl_2_ (1:1) and ultrasound. The combined extracts were evaporated under reduced pressure to give a greenish organic extract (38 g). The extract was subjected to flash RP18 silica gel column eluted with H_2_0 (A), H_2_O-CH_3_CN (2:8) (B), MeOH (C), and MeOH-CH_2_Cl_2_ (8:2) (D) to afford four fractions (A, B, C, and D). Then, fraction B (2 g) was subjected to flash RP18 silica gel column eluted with a solvent gradient of H_2_O-CH_3_CN to produce 12 fractions. Fraction 4 was subjected to HPLC purification (UP-50 DB.25M Uptisphere 5 µ) using 62% H_2_O-CH_3_CN at a flow rate of 3 mL/min to give acyclolaxaphycin B3 (3 mg, tr = 28.8 min) and acyclolaxaphycin B (2 mg, tr = 31.2 min).

### 3.3. Mass and NMR Spectroscopies

High-resolution ESI mass spectra were obtained using a Thermo Scientific LTQ Orbitrap mass spectrometer using electrospray ionization in positive mode. 1D-NMR and 2D-NMR experiments of synthetic compounds **1**, **2**, **8**, and **9** were acquired on a JEOL EX 400 spectrometer equipped with a NM-40 dual ^1^H, ^13^C probe, whereas those of natural compounds **9**, **10**, **11**, and **12** were acquired on a Brucker Avance 500 spectrometer equipped with a cryogenic probe (5 mm), all compounds in DMSO-*d*_6_ (500 μL) at 303 K. All chemical shifts were calibrated on the residual solvent peak (DMSO-*d*_6_, 2.50 ppm (^1^H) and 39.5 ppm (^13^C)). The chemical shifts, reported in delta (δ) units, and in parts per million (ppm) are referenced relatively to TMS.

### 3.4. Solid Phase Peptide Synthesis

Fmoc-protected amino acids, coupling reagents and Rink Amide resin were purchased from Novabiochem (Fontenay-sous-Bois, France) or Iris Biotech (Marktredwitz, Germany). Fmoc-(alloD)Ile-OH and Boc-(L)Lys(Fmoc)-OH were bought from Iris Biotech (Marktredwitz, Germany). DMF for peptide synthesis, and other chemicals were purchased from Aldrich (Saint-Quentin Fallavier, France). Automated solid phase Fmoc-synthesis was performed on a Liberty One synthesizer by CEM (Saclay, France).

**Synthesis protocol for automated solid phase peptide synthesis**: Automated solid-phase peptide synthesis was performed on a 0.1 mmol scale. 278 mg of Rink amide MBHA LL resin at a 0.36 mmol/g substitution level was pre-swollen for 60 min in DMF and the solvent was drained. Fmoc-removal was achieved with a 20% piperidine solution in DMF, initial deprotection 30 s, 40 W, 75 °C, second step 180 s, 40 W, 70 °C. After washing (3 × 7 mL DMF), 2.5 mL of a 0.2 M solution of the amino acid in DMF (5 eq relative to resin loading) was added. After addition of a 2 M solution of DIPEA in DMF (0.5 mL, 10 eq) and a 0.45 M solution of HATU in DMF (1 mL, 4.5 eq), the reaction solution was irradiated at 25 W for 5 min reaching a final temperature of 70 °C. All amino acids were coupled using these conditions except Fmoc-Gln(Trt)-OH for which a triple coupling was performed with a prolonged reaction time of 20 min. After full elongation of the peptide, the linear protected peptide linked to the resin was transferred into a batch reactor and the allyl protecting group was cleaved using Pd(P(Ph_3_))_4_ (m = 0.35 g, 0.3 mmol, 3 eq) with a solution of CHCl_3_/AcOH, NMM: 3.7/0.2/01 for 4 h at room temperature. After washing the resin using a solution of 0.5% diethyldithiocarbamate in DMF (3 × 10 mL) and DMF (3 × 10 mL) and lastly Fmoc removal, the cyclisation was performed with diisopropylcarbodiimide (DIC) and Oxyma, 1 mL, C = 0.5 M, 70° C 3 × 20 min, then 120 s at 20 °C between each cycle, followed by a final wash with DMF (3 × 7 mL). A Kaiser test was carried out in order to check reaction completion. Finally, the resin was washed with DCM (3 × 10 mL), transferred to a flask, and the cleavage cocktail (TFA, H_2_O, TIS 95/2.5/2.5) added. The resin was shaken for 3 h. The cleaving solution was collected and the resin washed with TFA (2 × 3 mL). The combined fractions were concentrated in vacuum. The crude peptide was precipitated in cold Et_2_O and finally centrifuged. The precipitate was washed with cold ether, extracted with water, and freeze-dried to yield the crude peptide.

**Analytical HPLC of peptides:** The HPLC analysis was run on a Waters (Guyancourt, France) 2695 HPLC system fitted with an ELS detector 2424 and a PDA 2998 using a Phenomenex Luna 3 μM C-8 column (150 × 3 mm). Eluents were water with 0.1% formic acid (buffer A) and acetonitrile with 0.1% formic acid (buffer B). Standard conditions comprised a flow rate of 0.4 mL/min eluting with 40% B to 100% B in 35 min. Standard conditions were applied to all HPLC analyses unless stated otherwise.

**Semi-preparative HPLC purification of peptide crudes:** A semi-preparative purification of cyclic peptides was performed using a Waters 1525 chromatography system fitted with a Waters 2487 tunable absorbance detector with detection at 214 nm (Guyancourt, France). Purification was performed by eluting solvents A (water) and B (acetonitrile) on a UP50DB C-18 column (250 × 10 mm) at 3 mL/min.

**LC-MS:** LC-MS analyses were carried out using a Thermo Fisher Scientific LC-MS device, Accela HPLC coupled to a LCQ Fleet equipped with an electrospray ionisation source and a 3D ion-trap analyzer (Villebon-sur-Yvette, France). The analysis was performed with a Phenomenex Kinetex C-18 column (100 × 300 mm) using gradient mixture of water with 0.1% formic acid (buffer A) and acetonitrile with 0.1% formic acid (buffer B). Standard conditions were a flow rate of 0.5 mL/min eluting with 20% B to 100% B in 15 min. Standard conditions were applied to all HPLC-MS analysis unless otherwise stated. 

**Figure 6:**
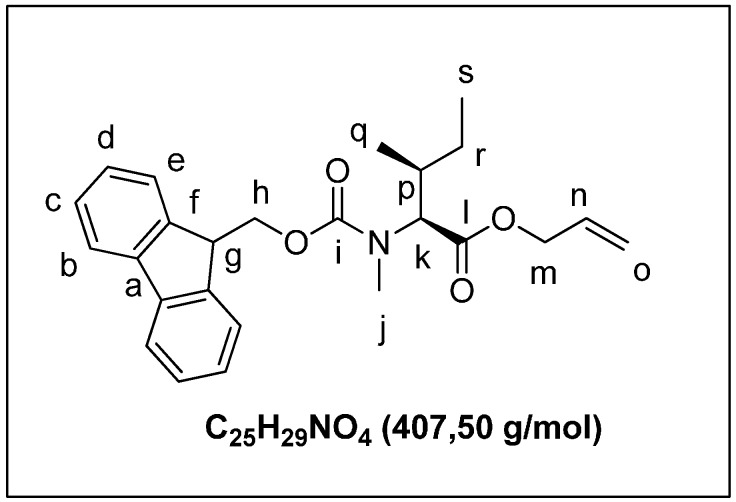
(2*S*,3*S*)-allyl 2-((((9H-fluoren-9-yl)methoxy)carbonyl)(methyl)amino)-3-methylpentanoate (**4a**).

Fmoc-NMeIle-OH (1.10 g, 3.0 mmol) was dissolved in DMF (7 mL) and cesium carbonate (0.98 g, 3.0 mmol) added. The mixture was stirred for 2 h. Allyl bromide (2.54 g, 1.80 mL, 21.0 mmol) was added to the mixture and stirring continued for 1 h resulting in a milky solution. The mixture was diluted to 20 mL with water and acidified with 1 M HCl. The aqueous layer was extracted with dichloromethane (25 mL, 3 × 25 mL). The organic layer was washed with brine (15 mL), dried over MgSO_4_, and filtered, and the solvent was removed under reduce pressure to give **4a** (Figure 6) as a colorless oil (0.92, 76%). ^1^H-NMR (CDCl_3_): 7.76 (d, 2H, *J* = 7.5 Hz); 7.59 (d, 2H_e_, *J* = 8.2 Hz); 7.39 (t, 2H_c_, *J* = 7.8 Hz); 7.28 (t, 2H_d_, *J* =7.5 Hz); 5.89 (m, 1H_n_); 5.32 (dt, 2H_m_, *J* = 17.4 Hz, *J* = 10.4 Hz); 4.61 (m, 2H_l_); 4.51 (m, 1H); 4.25 (m, 1H); 2.90 (s, 3H_j_); 2.00 (m, 1H_o_); 1.38 (m, 2H_r_); 0.90 (d, 3H_q_, *J* = 7.3 Hz); 0.85 (t, 3H_s_, *J* = 6.6 Hz). ^13^C-NMR (CDCl_3_): 171.6 (C_l_=O); 156.4 (C_i_=O); 144.1 (C_f_); 141.4 (C_a_); 131.8 (C_n_H); 128.4 (C_e_H) 127.7 (C_b_H); 125.1 (C_d_H); 120.0 (C_c_H); 118.5 (C_o_H2); 67.7 (C_k_H); 65.3 (C_h_H_2_); 62.5 (C_m_H_2_); 47.4 (C_g_H); 33.6 (C_j_H3); 30.1 (C_p_H); 25.0 (C_r_H_2_); 15.8 (C_q_H_3_); 11.0 (C_s_H_3_).

**Figure 7:**
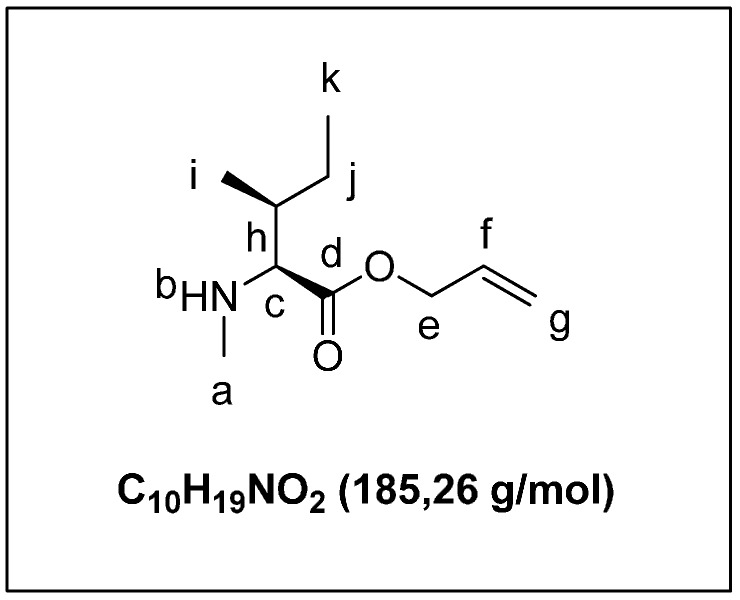
(2*S*,3*S*)-allyl 3-methyl-2-(methylamino)pentanoate (**4b**).

A solution of compound **4a** (1.10 g, 3.0 mmol) in DMF (5 mL) was treated with piperidine (1 mL). The mixture was stirred for 1 h. The mixture was diluted to 20 mL and the aqueous phase was extracted with cHex/AcOEt, 50/50 (3 × 30 mL). The organic layer was washed with brine (15 mL), dried over MgSO_4_, and filtered, and the solvent was removed under reduced pressure. The crude product was purified by flash chromatography on silica gel (eluent: cHex/AcOEt 6/4) to yield **4b** (Figure 7) as a colorless oil (0.29 g, 70%). ^1^H-NMR (CDCl_3_): 5.95 (m, 1H_f_); 5.32 (dd, 2H_g_, *J* = 22.5 Hz, *J* = 11.3 Hz); 4.65 (m, 2H_e_); 3.25 (d, 1H_c_, *J* = 5.3 Hz); 2.50 (3 s, 1H_a_); 1.92 (m, 1H_h_); 1.54 (m, 2H_j_); 0.92 (d, 3H_i_, *J* = 6.8 Hz); 0.80 (t, 3H_k_, *J* = 7.3 Hz). ^13^C-NMR (CDCl_3_): 170.6 (C_d_=O); 131.5 (C_f_H); 119.6 (C_g_H_2_); 66.9 (C_e_H_2_); 66.1 (C_c_H); 37.3 (C_h_H); 34.2 (C_a_H_3_); 26.3 (C_j_H_2_); 15.3 (C_i_H_3_); 11.7 (C_k_H_3_).

**Figure 8:**
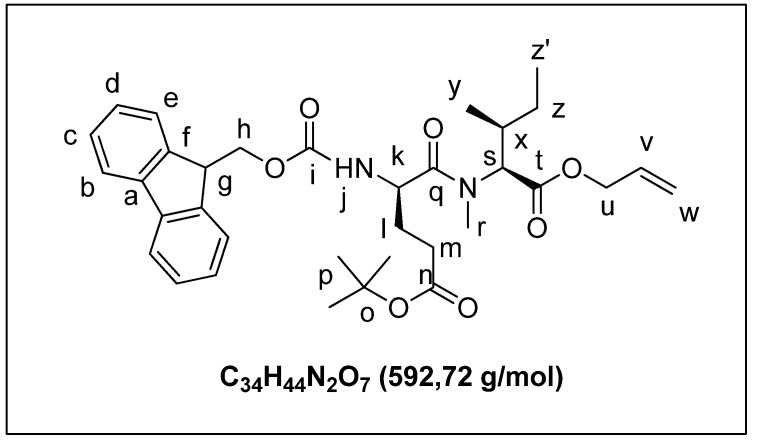
(*R*)-tert-butyl4-((((9H-fluoren-9-yl)methoxy)carbonyl)amino)-5-(((2*S*,3*S*)-1-(allyloxy)-3-methyl-1-oxopentan-2-yl)(methyl)amino)-5-oxopentanoate (**3a**).

A solution of Fmoc-Glu(OtBu)-OH (0.56 g, 1.30 mmol) in DMF (6 mL) was treated with DIEA (0.23 mL, 1.30 mmol). The mixture was cooled at 0 °C and COMU (0.56, 1.30 mmol) was added. After 5 min, solutions of compound **4b** (0.24 g, 1.30 mmol) in DMF (2 mL) and DIEA (0.23 mL, 1.30 mmol) were added successively at 0 °C. The reaction mixture was allowed to warm to room temperature and stirred for 48 h. The mixture was extracted with cHex/EtOAc, 50/50 (3 × 10 mL). The organic layer was washed successively with a solution of HCl 1M (10 mL), saturated aqueous NaHCO_3_ (10 mL), and brine (10 mL), and dried over MgSO_4_. The solvent was removed under reduced pressure. The crude product was purified by chromatography on silica gel using cHex/AcOEt (80/20) as eluent to yield compound **3a** (Figure 8) as a yellow oil (0.50 g, 70%). ^1^H-NMR (CDCl_3_): 7.75 (d, 2H_b_, *J* =7.3 Hz); 7.60 (d, 2H_e_, *J* = 6.2 Hz); 7.40 (t, 2Hc, *J* = 7.3 Hz); 7.22 (m, 2H_d_); 5.89 (m, NH_j_); 5.65 (m, 1H_v_); 5.32 (dd, 2H_w_, *J* = 15.6 Hz, *J* = 10.2 Hz); 4.97 (d, 1H_k_, *J* = 10.2 Hz); 4.75 (m, 1H_s_); 4.60 (m, 2H_u_); 4.36 (d, 2H_h_, *J* = 7.1 Hz); 4.21 (t, 1H_g_, *J* = 6.8 Hz); 3.10 (s, 3H_r_); 2.34 (m, 2H_m_); 2.03 (m, 2H_l_); 1.56 (m, 1H_x_); 1.45 (s, 9H_p_); 1.29 (m, 2H_z_); 0.98 (d, 3H_y_, *J* = 6.6 Hz); 0.86 (t, 3H_z’_, *J* = 7.3 Hz).

**Figure 9:**
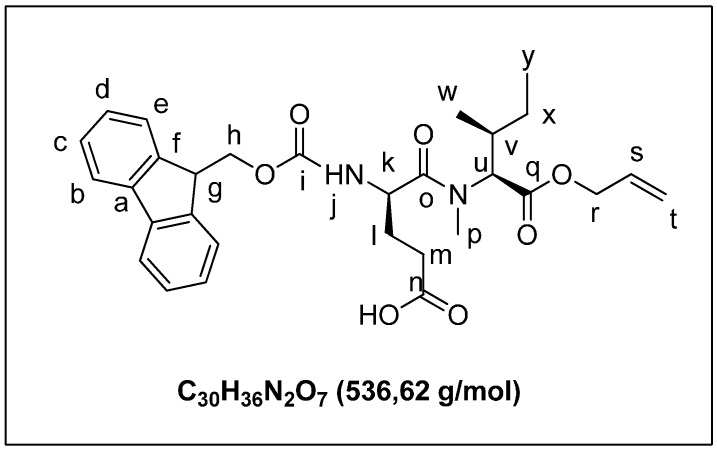
(*R*)-4-((((9H-fluoren-9-yl)methoxy)carbonyl)amino)-5-(((2*S*,3*S*)-1-(allyloxy)-3-methyl-1-oxopentan-2-yl)(methyl)amino)-5-oxopentanoic acid (**3b**).

A solution of compound **3a** (0.5 g, 0.90 mmol) in CH_2_Cl_2_ (4 mL) was treated with TFA (4 mL). The mixture was stirred for 12 h. The solvent was removed under reduced pressure and the crude product was purified by flash chromatography on silica gel (eluent: cHex/AcOEt 95/5) to yield **3b** (Figure 9) as a white solid (0.43 g, 96%). ^1^H-NMR (CDCl_3_): 7.75 (d, 2H_b_, *J* = 7.3 Hz); 7.60 (d, 2H_e_, *J* = 6.2 Hz); 7.40 (t, 2H_c_, *J* = 7.3 Hz); 7.22 (m, 2H_d_); 5.89 (m, NH_j_); 5.32 (dd, 2H_t_, *J* = 15.6 Hz, 10.2 Hz); 4.95 (d, 2H_k_, *J* = 7.2 Hz); 4.80 (m, 1H_u_); 4.60 (d, 2H_r_, *J* = 5.9 Hz); 4.36 (m, 2H_h_); 4.20 (t, 1H_g_, *J* = 7.0 Hz); 3.10 (s, 3H_p_); 2.47 (m, 2H_m_); 2.03 (m, 2H_l_); 1.56 (m, 1H_v_); 1.30 (m, 2H_x_); 0.98 (d, 3H_w_, *J* = 6.6 Hz); 0.86 (t, 3H_y_, *J* = 7.3 Hz). (ESI+) *m*/*z* [M + H]^+^ calcd for C_30_H_37_N_2_O_7_ 536.6, found 536.8.

## 4. Conclusions

As shown in this work, the preliminary synthesis of the simplified analogs (1) and (2) of the targeted natural compound was a prerequisite for the synthesis of ((2*S*,3*S*)-Hle^3^)laxaphycin B, the diastereoisomer of the natural compound that was initially described [9]. This initial work resulted in the development of a total synthesis of laxaphycin B and lyngbyacyclamide [19].

This methodological approach is also important for future synthesis of laxaB-type peptides such as the two new linear lipopeptides acyclolaxaphins B and B3 (11, 12) isolated from the tropical marine cyanobacterium *Anabaena* *cf.* *torulosa,* the same species in which we have already isolated the related cyclic peptides laxaphins B and B3. The presence of these acyclic laxa B-type compounds together with the cyclized ones in the same extract has, to our knowledge, never been described. The search of other minor acyclic potential biosynthetic precursors will provide valuable information concerning the hybrid PKS/NRPS biosynthetic pathway in this exciting lipophilic cyclic dodecapeptide series. Furthermore, these acyclic peptides suggest another possible disconnection for the future preparation of new analogs of laxaphycin B at the HLe^3^/Ala^4^ junction. In this case we could expect that the linear peptide would benefit from a pre-organized structure thanks to its almost syndiotactic character [37,38]. Further work is needed to verify this assumption and to develop a synthesis based on the use of a BAL linker [39]**,** which could enable other laxaphycin B type peptides to be synthesised.

## Supplementary Files

Supplementary File 1
