# Supplementary material for: Isolation and Synthesis of Laxaphycin B-Type Peptides: A Case Study and Clues to Their Biosynthesis
Source: Mar Drugs. 2015 Dec 5;13(12):7285–300. doi: 10.3390/md13127065 (PMC4699238; doi:10.3390/md13127065)
Supplement: Supplementary File 1 [file marinedrugs-13-07065-s001.docx]

Supplementary Materials: Isolation and Synthesis of Laxaphycin B-Type Peptides: A Case Study and Clues to Their Biosynthesis

Louis Bornancin ^1,†^, France Boyaud ^1,†^, Zahia Mahiout ^1^, Isabelle Bonnard ^1^, Suzanne C. Mills ^2,3^, Bernard Banaigs ^1,3,^*, and Nicolas Inguimbert ^1,^*

**Table S1.** ^1^H NMR data of synthetic compounds **1**, **2**, **8**, and **9** in DMSO-*d*_6_ (400 MHz).

| **Unit** | **Position** | **1** | **2** | **8** | **9** |
| --- | --- | --- | --- | --- | --- |
|  |  | **δ_H_[mult., *J* (Hz)]** | | | |
| β-Ala^1^/β-Ade^1^ | NH | 7.78 | 7.53 (d, 8.2) | 7.67 (d, 8.4) | 7.58 (d, 8.6) |
|  | CαH/H_2_ | 2.09 | 2.35/2.43 | 1.96/2.00 | 2.33/2.40 |
|  | CβH | 4.14 | 4.04 | 4.11 | 4.11 |
|  | CγH_2_ |  | 1.24/1.36 | 1.30 | 1.3/1.4 |
|  | CδH_2_ |  | 1.2 | 1.2 | 1.2 |
|  | CεH_2_ |  | 1.2 | 1.2 | 1.2 |
|  | CζH_2_ |  | 1.2 | 1.2 | 1.2 |
|  | CηH_2_ |  | 1.2 | 1.2 | 1.2 |
|  | CθH_2_ |  | 1.2 | 1.2 | 1.2 |
|  | CιH_3_ |  | 0.76 | 0.83 | 0.84 |
| Val^2^ | NH | 8.29 (d, 7.7) | 8.13 (d, 6,4) | 8.06 (d, 6.5) | 8.18 (d, 7.0) |
|  | CαH | 4.13 | 4.13 | 4.43 | 4.09 (d) |
|  | CβH_2_ | 2.09 | 2.06 | 1.96 | 1.97 (m) |
|  | CγH_3_ | 0.86 | 0.84 | 0.87 | 0.85 |
|  | Cγ'H_3_ | 0.86 | 0.84 | 0.87 | 0.91 |
| Thr^3^/Hle^3^ | NH | 7.82 |  | 8.0 (d, 8.4) | 7.94 (d, 8.0) |
|  | CαH | 4.15 |  | 4.21 (dd, 8.4/2.2) | 4.34 (dd, 9.1/2.0) |
|  | CβH | 4.02 |  | 3.48 | 3.49 |
|  | OH | - | 4.74 | 4.75 (d, 6.1) | 4.94 (d, 4.4) |
|  | CγH | 1.01 | 1.07 | 1.58 | 1.58 |
|  | CδH_3_ |  |  | 0.74 | 0.76 |
|  | Cδ'H_3_ |  |  | 0.91 | 0.89 |
| Ala^4^ | NH | 7.90 (d, 6.6) | 7.80 | 7.90 (d, 8.1) | 7.86 |
|  | CαH | 4.37 | 4.38 | 4.32 | 4.22 |
|  | CβH_3_ | 1.25 | 1.25 | 1.47 | 1.31 |
| Thr^5/^Hle^5^ | NH | 7.65 (d, 7.3) | 7.78 | 7.86 (d, 8.2) | 7.69 (d, 7.5) |
|  | CαH | 4.13 | 4.12 | 4.28 | 4.28 |
|  | CβH | 4.01 | 3.98 | 3.38 | 3.49 |
|  | OH | - | 4.98 | 4.87 | 5.03 |
|  | CγH | 1.01 | 0.99 | 1.75 | 1.56 |
|  | CδH_3_ |  |  | 0.81 | 0.76 |
|  | Cδ'H_3_ |  |  | 0.91 | 0.89 |
| Gln^6^ | NH | 7.78 | 7.68 (d, 7.9) | 7.71 (d, 7.6) | 7.77 (d, 7.4) |
|  | CαH | 4.64 | 4.63 | 4.58 | 4.63 |
|  | CβH_2_ | 1.70/1.89 | 1.75/1.89 | 1.77/1.90 | 1.75/1.97 |
|  | CγH_2_ | 2.06 | 2.06 | 2.15 | 2.04/2.10 |
|  | CONH_2_ | 6.83/7.22 | 6.85/7.22 | 6.90/7.21 | 6.85/7.22 |

**Table S1.** *Cont.*

| *N*-MeIle^7^ | NCH_3_ | 2.94 | 2.98 | | 2.98 | 2.97 |
| --- | --- | --- | --- | --- | --- | --- |
|  | CαH | 4.58 | 4.63 | | 4.71 (d, 11.0) | 4.72 (d, 11.0) |
|  | CβH | 1.90 | 1.90 | | 1.85 | 1.90 |
|  | CγH_2_ | 1.25 | 1.23 | | 1.25 | 0.89/1.29 |
|  | Cγ'H_3_ | 0.8 | 0.75 | | 0.74 | 0.76 |
|  | CδH_3_ | 0.9 | 0.78 | | 0.76 | 0.78 |
| Asn^8^/HAsn^8^ | NH | 8.23 (d, 7.5) | 8.23 (d, 6.7) | | 7.71 (d, 7.8) | 7.64 (d, 8.5) |
|  | CαH | 4.61 | 4.61 | 4.62 | | 4.63 |
|  | CβH | 2.41/2.51 | 2.41/2.50 | 4.34 | | 4.31 |
|  | OH |  |  | 5.80 (d, 6.5) | | 5.79 |
|  | CONH_2_ | 6.74/7.10 | 6.78/7.11 | 7.28 | | 7.27 |
| Thr^9^ | NH | 7.38 (d, 7.1) | 7.48 (d, 7.3) | 7.28 | | 7.33 (d, 8.0) |
|  | CαH | 4.32 | 4.40 | 4.45 | | 4.49 |
|  | CβH | 3.83 | 3.84 | 3.95 | | 3.95 |
|  | OH | - | 4.90 | 5.02 | | 4.94 |
|  | CγH_3_ | 1.08 | 1.06 | 1.06 | | 1.05 |
| Pro^10^/ Hyp^10^ | CαH | 4.35 | 4.29 | 4.34 | | 4.33 |
|  | CβH_2_ | 1.79/2.07 | 1.99/2.09 | 1.80/1.95 | | 1.82/2.04 |
|  | CγH_2_ | 1.88 | 1.75 | 1.90 | | 1.80/1.90 |
|  | CδH_2_ | 3.64/3.73 | 3.65/3.71 | 3.60 | | 3.68 |
| Leu^11^ | NH | 8.08 (d, 8.06) | 8.00 (d, 6.7) | 7.80 | | 7.89 |
|  | CαH | 4.41 | 4.38 | 4.10 | | 4.31 |
|  | CβH_2_ | 1.49 | 1.46 | 1.75 | | 1.47 |
|  | CγH | 1.50 | 1.55 | 1.50 | | 1.53 |
|  | CδH_3_ | 0.82 | 0.78 | 0.81 | | 0.82 |
|  | Cδ'H_3_ | 0.88 | 0.87 | 0.81 | | 0.87 |
| Thr^12^ | NH | 7.74 | 7.80 | 7.45 (d, 8.4) | | 7.74 |
|  | CαH | 4.12 | 4.03 | 4.00 | | 4.11 |
|  | CβH | 4.01 | 3.97 | 4.00 | | 4.00 |
|  | OH | - | 4.76 | 4.87 | | 4.78 |
|  | CγH_3_ | 1.10 | 0.99 | 0.99 | | 0.99 |

**Figure S1.** ^1^H-NMR and COSY spectra of laxaphycin B analog (**1**) in DMSO-*d*_6_ (400 MHz).

|  |
| --- |
|  |

**Figure S2.** ^1^H-NMR and COSY spectra of laxaphycin B analog (**2**) in DMSO-*d*_6_ (400 MHz).

|  |
| --- |
|  |

**Figure S3.** ^1^H and ^13^C NMR spectra of acyclolaxaphycin B (**11**) in DMSO-*d*_6_ (500 MHz, 303 K).

**Figure S4.** TOCSY spectrum of acyclolaxaphycin B (**11**) in DMSO-*d*_6_ (500 MHz, 303 K).

**Figure S5.** ROESY spectrum of acyclolaxaphycin B (**11**) in DMSO-*d*_6_ (500 MHz, 303 K).

**Figure S6.** HSQC spectrum of acyclolaxaphycin B (**11**) in DMSO-*d*_6_ (500 MHz, 303 K).

**Figure S7.** HSQC-TOCSY spectrum of acyclolaxaphycin B (**11**) in DMSO-*d*_6_ (500 MHz, 303 K).

**Figure S8.** HMBC spectrum of acyclolaxaphycin B (**11**) in DMSO-*d*_6_ (500 MHz, 303 K).

**Figure S9.** *Cont.*

**Figure S9.** ^1^H and ^13^C NMR spectra of acyclolaxaphycin B3 (**12**) in DMSO-*d*_6_ (500 MHz, 303 K).

**Figure S10.** TOCSY spectrum of acyclolaxaphycin B3 (**12**) in DMSO-*d*_6_ (500 MHz, 303 K).

**Figure S11.** ROESY spectrum of acyclolaxaphycin B3 (**12**) in DMSO-*d*_6_ (500 MHz, 303 K).

**Figure S12.** HSQC spectrum of acyclolaxaphycin B3 (**12**) in DMSO-*d*_6_ (500 MHz, 303 K).

**Figure S13.** HSQC-TOCSY spectrum of acyclolaxaphycin B3 (**12**) in DMSO-*d*_6_ (500 MHz, 303 K).

**Figure S14.** HMBC spectrum of acyclolaxaphycin B3 (**12**) in DMSO-*d*_6_ (500 MHz, 303 K).
